# Supplementary material for: Multi‐dataset identification of innovative feature genes and molecular mechanisms in keratoconus
Source: J Cell Mol Med. 2024 Sep 19;28(18):e70079. doi: 10.1111/jcmm.70079 (PMC11412914; doi:10.1111/jcmm.70079)
Supplement: Supplementary file 2 — Material S2. [file JCMM-28-e70079-s002.zip › 7.GSEA analysis/5.RNF217-AS1/KEGG_WNT_SIGNALING_PATHWAY.html]

Details for gene set KEGG\_WNT\_SIGNALING\_PATHWAY[GSEA]

|  || Dataset | RNF217-AS1.group.cls#h\_versus\_l |
| Phenotype | group.cls#h\_versus\_l |
| Upregulated in class | l |
| GeneSet | KEGG\_WNT\_SIGNALING\_PATHWAY |
| Enrichment Score (ES) | -0.3573691 |
| Normalized Enrichment Score (NES) | -1.5155654 |
| Nominal p-value | 0.012448133 |
| FDR q-value | 0.35399166 |
| FWER p-Value | 0.741 |
Table: GSEA Results Summary

  

Fig 1: Enrichment plot: KEGG\_WNT\_SIGNALING\_PATHWAY      
 Profile of the Running ES Score & Positions of GeneSet Members on the Rank Ordered List

  

| SYMBOL | TITLE | RANK IN GENE LIST | RANK METRIC SCORE | RUNNING ES | CORE ENRICHMENT || 1 | SFRP1 | na | 174 | 0.670 | 0.0272 | No |
| 2 | PRKCG | na | 203 | 0.657 | 0.0580 | No |
| 3 | NFATC2 | na | 219 | 0.642 | 0.0885 | No |
| 4 | DKK1 | na | 1281 | 0.411 | 0.0776 | No |
| 5 | SFRP4 | na | 1363 | 0.404 | 0.0947 | No |
| 6 | PPP3R2 | na | 1532 | 0.389 | 0.1086 | No |
| 7 | WNT9A | na | 2856 | 0.297 | 0.0846 | No |
| 8 | CAMK2A | na | 3603 | 0.267 | 0.0759 | No |
| 9 | WNT9B | na | 4560 | 0.231 | 0.0594 | No |
| 10 | CAMK2B | na | 5000 | 0.217 | 0.0572 | No |
| 11 | CHP2 | na | 5296 | 0.206 | 0.0586 | No |
| 12 | WIF1 | na | 5471 | 0.201 | 0.0632 | No |
| 13 | WNT8A | na | 5716 | 0.192 | 0.0654 | No |
| 14 | CER1 | na | 6743 | 0.160 | 0.0434 | No |
| 15 | RAC3 | na | 7163 | 0.148 | 0.0385 | No |
| 16 | PLCB3 | na | 7444 | 0.141 | 0.0372 | No |
| 17 | PPARD | na | 8362 | 0.120 | 0.0164 | No |
| 18 | SIAH1 | na | 8692 | 0.112 | 0.0123 | No |
| 19 | CSNK2A1 | na | 9124 | 0.103 | 0.0048 | No |
| 20 | PPP2R1B | na | 9136 | 0.102 | 0.0094 | No |
| 21 | PSEN1 | na | 9208 | 0.101 | 0.0122 | No |
| 22 | DKK4 | na | 9421 | 0.097 | 0.0107 | No |
| 23 | WNT3 | na | 9757 | 0.090 | 0.0054 | No |
| 24 | CTBP2 | na | 10134 | 0.084 | -0.0014 | No |
| 25 | ROCK1 | na | 10313 | 0.081 | -0.0027 | No |
| 26 | CSNK1A1 | na | 10620 | 0.076 | -0.0079 | No |
| 27 | GSK3B | na | 10962 | 0.071 | -0.0143 | No |
| 28 | WNT2 | na | 11310 | 0.066 | -0.0212 | No |
| 29 | PPP2R5B | na | 11671 | 0.060 | -0.0287 | No |
| 30 | NFATC1 | na | 11693 | 0.060 | -0.0264 | No |
| 31 | FZD9 | na | 12064 | 0.055 | -0.0345 | No |
| 32 | PPP2R5C | na | 12306 | 0.051 | -0.0390 | No |
| 33 | CXXC4 | na | 12542 | 0.048 | -0.0435 | No |
| 34 | MAPK9 | na | 12895 | 0.044 | -0.0516 | No |
| 35 | NFAT5 | na | 12924 | 0.043 | -0.0503 | No |
| 36 | TCF7L2 | na | 12940 | 0.043 | -0.0487 | No |
| 37 | RAC2 | na | 12945 | 0.043 | -0.0467 | No |
| 38 | FOSL1 | na | 12974 | 0.042 | -0.0455 | No |
| 39 | SFRP5 | na | 12995 | 0.042 | -0.0440 | No |
| 40 | SFRP2 | na | 13115 | 0.041 | -0.0455 | No |
| 41 | DAAM2 | na | 13651 | 0.034 | -0.0593 | No |
| 42 | PPP3CB | na | 13986 | 0.031 | -0.0675 | No |
| 43 | DVL1 | na | 14350 | 0.026 | -0.0767 | No |
| 44 | TBL1XR1 | na | 14525 | 0.024 | -0.0806 | No |
| 45 | CREBBP | na | 14557 | 0.024 | -0.0803 | No |
| 46 | SMAD3 | na | 14794 | 0.021 | -0.0861 | No |
| 47 | DAAM1 | na | 14831 | 0.021 | -0.0862 | No |
| 48 | PPP2R5A | na | 14850 | 0.021 | -0.0857 | No |
| 49 | RHOA | na | 14935 | 0.020 | -0.0872 | No |
| 50 | WNT3A | na | 14964 | 0.019 | -0.0870 | No |
| 51 | CTBP1 | na | 15097 | 0.018 | -0.0900 | No |
| 52 | AXIN1 | na | 15329 | 0.016 | -0.0959 | No |
| 53 | PPP2CB | na | 15463 | 0.014 | -0.0991 | No |
| 54 | MAPK10 | na | 15620 | 0.013 | -0.1030 | No |
| 55 | CHD8 | na | 15663 | 0.012 | -0.1036 | No |
| 56 | DVL3 | na | 15800 | 0.011 | -0.1070 | No |
| 57 | CSNK1A1L | na | 15884 | 0.010 | -0.1089 | No |
| 58 | CAMK2G | na | 16021 | 0.008 | -0.1125 | No |
| 59 | SMAD2 | na | 16024 | 0.008 | -0.1121 | No |
| 60 | WNT4 | na | 16250 | 0.006 | -0.1183 | No |
| 61 | RBX1 | na | 16317 | 0.006 | -0.1199 | No |
| 62 | FBXW11 | na | 16324 | 0.006 | -0.1198 | No |
| 63 | TCF7L1 | na | 16424 | 0.004 | -0.1225 | No |
| 64 | PPP2R5D | na | 16548 | 0.003 | -0.1259 | No |
| 65 | SKP1 | na | 16603 | 0.003 | -0.1273 | No |
| 66 | MAPK8 | na | 16681 | 0.002 | -0.1295 | No |
| 67 | SMAD4 | na | 16849 | 0.000 | -0.1343 | No |
| 68 | PPP2R5E | na | 17233 | -0.004 | -0.1452 | No |
| 69 | PPP2R1A | na | 17564 | -0.007 | -0.1544 | No |
| 70 | WNT1 | na | 17572 | -0.007 | -0.1542 | No |
| 71 | LRP6 | na | 18049 | -0.011 | -0.1675 | No |
| 72 | CSNK2A2 | na | 18151 | -0.012 | -0.1698 | No |
| 73 | RAC1 | na | 18357 | -0.014 | -0.1751 | No |
| 74 | FZD5 | na | 18663 | -0.017 | -0.1831 | No |
| 75 | ROCK2 | na | 18737 | -0.018 | -0.1843 | No |
| 76 | CCND1 | na | 18820 | -0.019 | -0.1858 | No |
| 77 | PORCN | na | 19233 | -0.022 | -0.1966 | No |
| 78 | WNT10A | na | 19364 | -0.024 | -0.1992 | No |
| 79 | BTRC | na | 19413 | -0.024 | -0.1995 | No |
| 80 | CSNK2B | na | 19468 | -0.025 | -0.1998 | No |
| 81 | FZD6 | na | 19485 | -0.025 | -0.1991 | No |
| 82 | PPP2CA | na | 19715 | -0.027 | -0.2044 | No |
| 83 | CTNNB1 | na | 19818 | -0.028 | -0.2060 | No |
| 84 | FRAT2 | na | 19829 | -0.028 | -0.2049 | No |
| 85 | TCF7 | na | 19939 | -0.029 | -0.2066 | No |
| 86 | WNT7B | na | 19942 | -0.029 | -0.2053 | No |
| 87 | CAMK2D | na | 20347 | -0.033 | -0.2154 | No |
| 88 | MAP3K7 | na | 20426 | -0.034 | -0.2160 | No |
| 89 | CTNNBIP1 | na | 20657 | -0.036 | -0.2209 | No |
| 90 | PPP3R1 | na | 20845 | -0.038 | -0.2244 | No |
| 91 | NKD2 | na | 21081 | -0.041 | -0.2293 | No |
| 92 | CHP1 | na | 21142 | -0.042 | -0.2290 | No |
| 93 | LRP5 | na | 21258 | -0.043 | -0.2303 | No |
| 94 | VANGL1 | na | 21304 | -0.043 | -0.2295 | No |
| 95 | PLCB4 | na | 21374 | -0.044 | -0.2294 | No |
| 96 | WNT7A | na | 21895 | -0.050 | -0.2420 | No |
| 97 | CUL1 | na | 21972 | -0.050 | -0.2418 | No |
| 98 | NKD1 | na | 22010 | -0.051 | -0.2404 | No |
| 99 | SENP2 | na | 22858 | -0.060 | -0.2620 | No |
| 100 | PRKACA | na | 23066 | -0.063 | -0.2650 | No |
| 101 | EP300 | na | 23570 | -0.069 | -0.2762 | No |
| 102 | WNT10B | na | 23653 | -0.070 | -0.2752 | No |
| 103 | PRKCA | na | 23805 | -0.072 | -0.2761 | No |
| 104 | PPP3CA | na | 24064 | -0.076 | -0.2798 | No |
| 105 | NFATC3 | na | 24080 | -0.076 | -0.2766 | No |
| 106 | CACYBP | na | 24196 | -0.078 | -0.2762 | No |
| 107 | WNT2B | na | 24447 | -0.082 | -0.2795 | No |
| 108 | WNT5A | na | 25202 | -0.094 | -0.2968 | No |
| 109 | FZD10 | na | 25265 | -0.095 | -0.2940 | No |
| 110 | TBL1Y | na | 25836 | -0.104 | -0.3055 | No |
| 111 | NLK | na | 26012 | -0.107 | -0.3054 | No |
| 112 | PRKACB | na | 26457 | -0.116 | -0.3126 | No |
| 113 | CCND3 | na | 26722 | -0.120 | -0.3145 | No |
| 114 | DVL2 | na | 26784 | -0.122 | -0.3104 | No |
| 115 | PPP3CC | na | 26944 | -0.124 | -0.3090 | No |
| 116 | TBL1X | na | 27026 | -0.126 | -0.3053 | No |
| 117 | JUN | na | 27259 | -0.131 | -0.3057 | No |
| 118 | FZD1 | na | 27779 | -0.143 | -0.3138 | No |
| 119 | WNT8B | na | 29287 | -0.184 | -0.3485 | Yes |
| 120 | RUVBL1 | na | 29480 | -0.190 | -0.3449 | Yes |
| 121 | NFATC4 | na | 29771 | -0.199 | -0.3437 | Yes |
| 122 | WNT16 | na | 30080 | -0.209 | -0.3425 | Yes |
| 123 | CCND2 | na | 30134 | -0.211 | -0.3339 | Yes |
| 124 | MYC | na | 30784 | -0.234 | -0.3414 | Yes |
| 125 | FRAT1 | na | 30893 | -0.238 | -0.3331 | Yes |
| 126 | FZD4 | na | 30972 | -0.241 | -0.3237 | Yes |
| 127 | FZD3 | na | 31028 | -0.243 | -0.3136 | Yes |
| 128 | PLCB2 | na | 31149 | -0.248 | -0.3052 | Yes |
| 129 | VANGL2 | na | 31240 | -0.251 | -0.2957 | Yes |
| 130 | APC | na | 31337 | -0.256 | -0.2861 | Yes |
| 131 | PRKX | na | 31362 | -0.257 | -0.2744 | Yes |
| 132 | FZD8 | na | 31536 | -0.265 | -0.2667 | Yes |
| 133 | WNT6 | na | 31862 | -0.283 | -0.2624 | Yes |
| 134 | SOX17 | na | 31965 | -0.289 | -0.2515 | Yes |
| 135 | TP53 | na | 32227 | -0.304 | -0.2443 | Yes |
| 136 | DKK2 | na | 32234 | -0.305 | -0.2298 | Yes |
| 137 | PRICKLE1 | na | 32368 | -0.315 | -0.2185 | Yes |
| 138 | PLCB1 | na | 32371 | -0.315 | -0.2034 | Yes |
| 139 | PRICKLE2 | na | 33101 | -0.368 | -0.2068 | Yes |
| 140 | PRKACG | na | 33174 | -0.374 | -0.1909 | Yes |
| 141 | FZD7 | na | 33284 | -0.383 | -0.1756 | Yes |
| 142 | FZD2 | na | 33686 | -0.431 | -0.1664 | Yes |
| 143 | LEF1 | na | 34024 | -0.487 | -0.1527 | Yes |
| 144 | APC2 | na | 34163 | -0.517 | -0.1318 | Yes |
| 145 | PRKCB | na | 34175 | -0.520 | -0.1071 | Yes |
| 146 | WNT5B | na | 34219 | -0.533 | -0.0827 | Yes |
| 147 | MMP7 | na | 34446 | -0.621 | -0.0593 | Yes |
| 148 | WNT11 | na | 34469 | -0.631 | -0.0296 | Yes |
| 149 | AXIN2 | na | 34629 | -0.769 | 0.0029 | Yes |
Table: GSEA details [plain text format]

  

Fig 2: KEGG\_WNT\_SIGNALING\_PATHWAY      
 Blue-Pink O' Gram in the Space of the Analyzed GeneSet

  

Fig 3: KEGG\_WNT\_SIGNALING\_PATHWAY: Random ES distribution      
 Gene set null distribution of ES for **KEGG\_WNT\_SIGNALING\_PATHWAY**
